# Predicted Functional Implications of Phosphorylation of Regulator of G Protein Signaling Protein in Plants

**DOI:** 10.3389/fpls.2017.01456

**Published:** 2017-08-25

**Authors:** Meral Tunc-Ozdemir, Bo Li, Dinesh K. Jaiswal, Daisuke Urano, Alan M. Jones, Matthew P. Torres

**Affiliations:** ^1^Department of Biology, University of North Carolina at Chapel Hill, Chapel Hill NC, United States; ^2^Temasek Life Sciences Laboratory, National University of Singapore Singapore, Singapore; ^3^Department of Pharmacology, University of North Carolina at Chapel Hill, Chapel Hill NC, United States; ^4^School of Biological Sciences, Georgia Institute of Technology, Atlanta GA, United States

**Keywords:** Arabidopsis Regulator of G Signaling protein 1 (AtRGS1), the leucine-rich repeat receptor-like kinase (LRR RLK), BRI1-associated receptor kinase (BAK1), brassinosteroid insensitive1 (BRI1) -LIKE 3 (BRL3), AtPep1 receptor 1 (PEPR1), with no lysine kinase (WNK), post-translational modification (PTM), modified alignment positions (MAP)

## Abstract

Heterotrimeric G proteins function in development, biotic, and abiotic stress responses, hormone signaling as well as sugar sensing. We previously proposed that discrimination of these various external signals in the G protein pathway is accomplished in plants by membrane-localized receptor-like kinases (RLKs) rather than G-protein-coupled receptors. *Arabidopsis thaliana* Regulator of G Signaling protein 1 (AtRGS1) modulates G protein activation and is phosphorylated by several RLKs and by WITH-NO-LYSINE kinases (WNKs). Here, a combination of *in vitro* kinase assays, mass spectrometry, and computational bioinformatics identified and functionally prioritized phosphorylation sites in AtRGS1. Phosphosites for two more RLKs (BRL3 and PEPR1) were identified and added to the AtRGS1 phosphorylation profile. Bioinformatics analyses revealed that RLKs and WNK kinases phosphorylate plant RGS proteins within regions that are conserved across eukaryotes and at a high frequency. Four phospho-sites among 14 identified are proximal to equivalent mammalian phosphosites that impact RGS function, including: pS437 and pT267 in GmRGS2, and pS339 and pS436 in AtRGS1. Based on these analyses, we propose that pS437 and pS436 regulate GmRGS2 and AtRGS1 protein interactions and/or localization, whereas pT267 is important for modulation of GmRGS2 GAP activity and localization. Moreover, pS339 most likely affects AtRGS1 activation.

## Introduction

In *Arabidopsis thaliana*, the heterotrimeric G protein complex contains only one canonical Gα subunit (AtGPA1), one Gβ, and three Gγ subunits (Urano et al., 2013). Paradoxically, this small number of complex couples numerous extracellular signals to cytoplasmic changes (Joo et al., 2005; Booker et al., 2012; Jiang et al., 2012; Lee et al., 2013; Colaneri et al., 2014; Xu et al., 2015). Heterotrimeric G proteins control growth, cell proliferation, pathogen defense, stomata movements, channel regulation, sugar sensing, and some hormone responses (Urano et al., 2013). In animal cells, the heterotrimeric G protein complex is an on-off switch regulated by plasma membrane receptors. Once bound by the ligand, these so-called G-protein-coupled receptors (GPCRs) catalyze the exchange of guanine nucleotide GDP for GTP which then activates the G protein. In plant cells, the Gα protein spontaneously binds GTP, therefore there is no need for GPCRs. Instead, most plant G proteins are kept in an inactive (GDP-bound) state by a receptor-like inhibitor and/or by a yet-to-be identified regulator. The prototype of the receptor-like inhibitor is Arabidopsis Regulator of G Signaling protein 1 (AtRGS1). AtRGS1 has an N-terminal seven-transmembrane domain and a cytoplasmic C-terminal catalytic RGS domain. Expression of AtRGS1 complemented the pheromone super-sensitivity phenotype of a yeast RGS mutant, sst2Δ (Chen et al., 2003) showing the functional relevance of AtRGS1 beyond plant cells. AtRGS1 modulates G signaling in a manner induced by signals such as glucose (Urano et al., 2012) and the pathogen-associated molecular pattern (PAMP) 22-amino acid peptide, flg22 (Tunc-Ozdemir et al., 2016). Phosphorylation of AtRGS1 by various kinases is essential for its endocytosis thus activation of G protein signaling (Urano et al., 2012; Fu et al., 2014; Tunc-Ozdemir et al., 2016).

Recent studies on direct activation of G signaling by receptor-like kinases (RLK) in plants (Choudhury and Pandey, 2015; Tunc-Ozdemir et al., 2016) revealed a previously unknown signal transduction pathway whereby an elicitor of the immune response, flg22, induces interaction between BAK1 and AtRGS1. This consequently leads to AtRGS1 endocytosis and physical uncoupling between AtGPA1 and AtRGS1. Within this pathway, AtRGS1 serves as a ligand-dependent signal modulator of heterotrimeric G protein signaling, but in a manner that depends on its phosphorylation by RLKs (Tunc-Ozdemir et al., 2016; Tunc-Ozdemir and Jones, 2017b). Included among the list of responsible RLKs is BRI1-associated receptor kinase (BAK1), which is the co-receptor required for signal transduction in PAMP-triggered immunity, cell death, and development (Li et al., 2002; He et al., 2007; Schulze et al., 2010). Similarly, RLK-mediated phosphorylation of *Glycine max* RGS2 (GmRGS2) by Nod factor receptor 1 (NFR1), a LysM receptor kinase (Choudhury and Pandey, 2015) was shown to be involved in nodule development.

The Arabidopsis genome has more than 200 LRR RLK subfamily members that regulate developmental and defense-related processes including cell proliferation, stem cell maintenance, hormone perception, host-specific as well as non-host-specific defense response, wounding response, and symbiosis (Torii, 2004). Recent evidence suggests that AtRGS1 serves as a substrate for RLKs involved in growth, development, innate immunity, cell death, and development (Tunc-Ozdemir et al., 2016). Some of these leucine-rich repeat receptor-like kinases (LRR RLK) are brassinosteroid insensitive1-like 3 (BRL3), Somatic embryogenesis like kinase 3 (SERK3)/BAK1, and PEP1 receptor 1 (PEPR1). These kinases phosphorylate AtRGS1 at its C terminus (Tunc-Ozdemir et al., 2016) *in vitro*. BRL3 and AtRGS1 function together to fine tune growth inhibition and ROS activation (Tunc-Ozdemir and Jones, 2017a) whereas BAK1 interacts with AtRGS1 in PAMP response. In addition to RLKs, AtRGS1 is phosphorylated at Ser by AtWNK8 and AtWNK1, which are two of 10 WNK (WITH NO LYSINE) family Ser/Thr kinases important for sugar signaling, salt, and osmotic stresses and flowering in Arabidopsis (Tsuchiya and Eulgem, 2010; Zhang et al., 2013; Fu et al., 2014).

Receptor-like kinases also interact directly with heterotrimeric G protein components in plants. For example, Arabidopsis *zygotic arrest 1* (*ZAR1*) encodes a member of the RLK/Pelle kinase family that physically interacts with the heterotrimeric G protein Gβ to regulate the division of zygote and the cell fate of its daughter cells (Yu et al., 2016). Physical interaction between the Gα, Gγ1 and Gγ2 subunits, and the defense-related RLKs chitin elicitor receptor kinase 1 (CERK1), BAK1 and BIR1 suggests that heterotrimeric G proteins mediate signal transduction immediately downstream of the RLKs (Aranda-Sicilia et al., 2015), which also have demonstrated roles in plant pathogenesis. The non-canonical Gα protein XLG2 directly interacts with plasma membrane localized RLK, FLAGELLIN-SENSING 2 (FLS2) and cytoplasmic kinase BIK1 (Liang et al., 2016). Studies on stem cell proliferation through CLAVATA signaling in Arabidopsis (Ishida et al., 2014) and in maize (Bommert et al., 2013) showed physical interactions between AGB1 and RPK2 and Gα protein (Ct2) and Fea2 (CLAVATA-2), respectively. Finally, genetic evidence suggests that some RLKs may also serve as receptors or co-receptors in G-protein-coupled signaling in plants (Lease et al., 2001; Llorente et al., 2005; Zhang et al., 2008; Bommert et al., 2013; Liu et al., 2013; Ishida et al., 2014). In summary, RLKs are clearly ensconced in G-protein signaling in plants.

We mapped additional RLK transphosphorylation sites on the AtRGS1 protein by high-resolution mass spectrometry and, by including known phospho-sites, we used a quantitative post-translational modification (PTM) informatics method – SAPH-ire – to enable quantitative analysis of PTM hotspots in protein families. Unlike traditional methods of PTM analysis, SAPH-ire comprehensively integrates all modification data within a protein family, a method that has been validated to improve functional prioritization of PTMs (Dewhurst et al., 2015; Torres et al., 2016; Dewhurst and Torres, 2017). Analysis of RGS hotspots across the mammalian and plant proteome resulted in the discovery of phosphorylation sites that are prioritized according to their relative importance in different functions. The pS437 and pT267 that were found in GmRGS2 previously (Choudhury and Pandey, 2015) and pS339 that we found in AtRGS1 were suggested to be functional due to their close location to experimentally proven PTMs changing non-plant RGS proteins’ activity, localization or stability. The pS437 and pT267 of GmRGS2 are reported to be important for GTPase-Accelerating activity along with four other phosphorylation sites (Choudhury and Pandey, 2015) thus validating our analyses. We propose that GmRGS2 pS269, pS277, pS405 and AtRGS1 pS339, pS365, pT375, pT379, pS417, and pS453 are involved in protein–protein interactions because they reside on a protein interface. The highly conserved GmRGS2 S437, T428, and AtRGS1 S428, S435/436 residues are part of a phosphorylation island suggested to be important for regulation of RGS protein interactions or its localization. Previous reports showing phosphorylation of AtRGS1 S428, S435/436 to be necessary for protein trafficking (Urano et al., 2012; Fu et al., 2014; Tunc-Ozdemir et al., 2016) support this finding. Here, we describe two new AtRGS1 kinases and mapped the phosphorylated residues. We combined all the data on Arabidopsis and soybean RGS protein phosphorylation and subjected it to a new bioinformatic method that enabled us to speculate on the functions of plant RGS PTMs and to prioritize them for testing experimentally in the future.

## Materials and Methods

### Protein Purification and *In Vitro* Phosphorylation Assay

Complementary DNAs encoding the complete cytoplasmic domain (juxtamembrane region, catalytic kinase domain, and C-terminal region) for BRL3 (amino acids 773–1164) and PEPR1 (amino acids 770–1123) were cloned into a modified pET Gateway vector for expression of His-tagged recombinant protein in *Escherichia coli* BL21 (DE3) pLysS cells (Mitra et al., 2015). BRL3 and PEPR1 purification was performed according to Tunc-Ozdemir et al. (2016). His6-tagged RGS1 cytoplasmic region was prepared as described (Urano et al., 2012). Purified kinase proteins were mixed with 6XHis-tagged AtRGS1 C-terminal domain (His6-RGS-J5: AtRGS1-coding sequences RGS box + Ct [amino acids 284–459]) protein in 25 μl of reaction buffer; 50 mM Tris-HCl (pH 7.5), 10 mM MgCl_2_, 10 mM MnCl_2_, 1 mM dithiothreitol, 1 μg/ml leupeptin, 0.1 μM calyculin A and 50 μM ATP (including 2 μCi radio-labeled [γ-32P] ATP at 3,000 Ci/mmol), and then incubated at room temperature for 8.5 h to achieve saturation. Approximately, 1 μg of kinase domain and 2.7 μg of His6-RGS-J5 were added into each reaction. The reaction was stopped by adding 10 μl of 5x Laemmli sample buffer. The kinase and His6-RGS-J5 proteins were separated on a SDS-PAGE gel, and the radio-labeled phosphate transferred on proteins was visualized with a phospho image analyzer.

### SAPH-ire

Data collection, correction, and processing with SAPH-ire was performed as previously described (Dewhurst et al., 2015; Torres et al., 2016) with the exception that the SAPH-ire-generated RGS family alignment (PTM sub-alignment; IPR001617) was expanded to include AtRGS1 and GmRGS2 as well as the latest PTM dataset from dbPTM (Huang et al., 2016). From this expanded table, we quantified the following features: phospho-acceptor residue conservation (conservation of serine, threonine, or tyrosine in an alignment position), total residue conservation (conservation of the most frequently occurring residue in an alignment position), membership (non-gap percentage in the alignment position), PTM count (count of experimentally observed PTMs within the alignment position), known function classification (binary classifier indicating whether there is at least one published piece of evidence for PTM functionality), cluster PTM count (the count of observed PTMs within ±2 positions of the alignment position), interface residence (binary classifier indicating whether the alignment position contains any residues known to be at a protein–protein interface based on crystallographic evidence). To determine the total residue and phospho-acceptor residue conservation values for plant-specific 7TM-RGS proteins, we established a multiple sequence alignment (MSA) of AtRGS1, GmRGS2 and all other known 7TM-RGS proteins. The sequences were selected to maximize coverage and minimize bias across archaeplastida given the available genomes. *Amborella* was chosen because of its position at the base of the angiosperms. *Pinus, Selaginella*, and *Chara* were included as divergent sequences outside the angiosperm, represented by a gymnosperm, a lycophyte, and a green alga, respectively. There are few monocot genomes available outside the cereals but the available five non-cereal monocots were included. The remaining were eudicots covering six classes. The plant sub-alignment profile consisting of AtRGS1 and GmRGS2 was then extracted from this alignment and included in a profile-to-profile alignment with the PTM sub-alignment from SAPH-ire, which is restricted to family members with at least one PTM and/or a high-resolution 3-D structure. MSAs were generated with MUSCLE using default parameters (Edgar, 2004). Phylogenetic analysis was accomplished using Unipro UGENE (Okonechnikov et al., 2012). Data for functional phosphosites were retrieved from Phosphosite Plus (Hornbeck et al., 2014). Statistical and graphic analyses were performed using JMP 12.1 (SAS, Inc.).

### Structural Homology Modeling and Projection of PTMs

Structural homology modeling of the RGS domain of AtRGS1 was carried out with the website SWISS-MODEL, which identified the PDB structure 2GTP (human RGS1 bound to activated Gαi1) as the most appropriate homology target for AtRGS1 (PMID 16301204). Next, we used the 2GTP structure as a template for alignment of the RGS domain model and AtGPA1 (PDB: 2XTZ). Interface residues were identified within four angstroms using PyMol (The PyMOL Molecular Graphics System, Version 1.2r3pre, Schrödinger, LLC). Interface data (binary classified as 0 or 1) were tabulated with PTM feature data from SAPH-ire and an Integrating Score calculated as follows to enable comprehensive relation of all features for each RGS domain MAP follows:

IS = (CPC) ^∗^ (PC) ^∗^ (PRC) ^∗^ W_1_(NKC) ^∗^ W_2_(PPI)

*where*,

      IS = Integrative score

      CPC = Cluster PTM count

      PC = PTM count

      PRC = PTM residue conservation

      NKC = Neighbor known function count

      PPI = Protein–protein interface residence

      W1 = Conditional weight factor 1

      W2 = Conditional weight factor 2.

Each feature was chosen based on extensive modeling of the relationship between MAP features and biological functionality for PTMs (Torres et al., 2016; Dewhurst and Torres, 2017). The resulting scores were organized into bins and used for color coding residues in the domain structure or for rank ordering.

## Results and Discussion

### The C-Terminal Tail of AtRGS1 Is Phosphorylated by BRL3 and PEPR1 *In Vitro*

**FIGURE 1:**
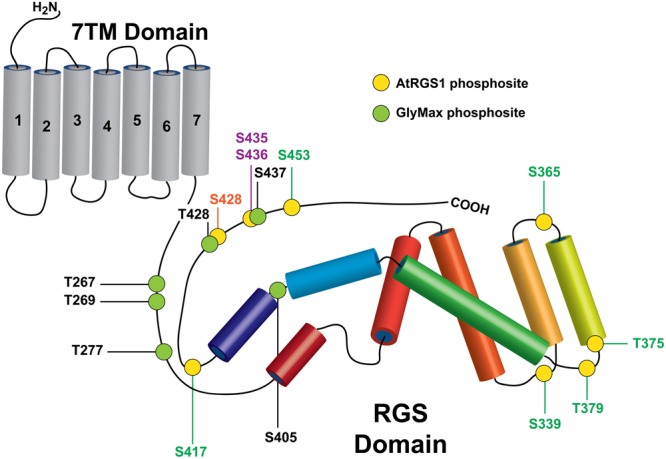
Rreceptor-like kinase (RLK) targets on plant RGS proteins. Phosphorylation sites identified in this study on AtRGS1 and GmRGS2 as well as sites identified previously are illustrated in the context of the domain architectures of AtRGS1 and GmRGS2 (yellow and green circles). Kinases are indicated by site ID color: BRL3 (green), BAK1, PEPR1, and WNK8 (orange) and WNK8 only (purple) for both RGS proteins. NRF1 phosphorylation sites are indicated for GmRGS2 sites (black). Domain sizes are not to scale to emphasize RGS domain and surrounding regions.

A screen of 70 active, recombinant arginine–aspartate type LRR RLKs revealed that more than 10 RLKs phosphorylate AtRGS1 *in vitro* including BAK1, BRL3, and PEPR1 (belonging to LRR II, X, and XI Subfamilies of Arabidopsis LRR respectively) (Tunc-Ozdemir et al., 2016). Although *in vitro* phosphorylation of AtRGS1 by BRL3 was previously shown, the BRL3 and PEPR1 transphosphorylation sites on AtRGS1 were not mapped through high-resolution mass spectrometry. Therefore, 6XHis-tagged AtRGS1 box + C-terminal domain recombinant protein phosphorylated by purified BRL3 and PEPR1 kinase domains were analyzed by liquid chromatography–mass spectrometry (LC–MS). The peptide mass tolerance was set as 100 ppm and 0.02 Da for MS/MS tolerance. Sequence coverage identified by mass spectrometry and *in vitro* kinase reactions are demonstrated in **Supplementary Figure S1**. As summarized in **Figure 1**, the identified AtRGS1 phosphorylation sites (S339, S365, T375, T379, S417, S428, and S453) are listed along with previously reported WNK8-mediated phosphorylation of AtRGS1 (S428, S436) and NFR1-mediated phosphorylation sites on GmRGS2 (T267, S269, S277, S405, T428, and T437).

### Analysis of Plant RGS Phosphorylation Sites by Comparison with Non-plant RGS Proteins

Long-standing evidence from animal systems revealed that RGS proteins are prominent targets of PTM. In many cases, site-specific phosphorylation of animal RGS proteins has also been shown to serve a regulatory function (Kach et al., 2012). In contrast, phosphorylation studies in 7TM-RGS proteins from plants are sparse and their functional significance less well-understood.

Several common features (i.e., characteristics of modified residues) were shown to be predictive for phosphorylation sites that are regulatory for specific protein families. These include but are not limited to several features derived from MSA of modified proteins, including phospho-acceptor residue conservation, phosphorylation observation frequency (PTM count), phosphorylation site clustering, among others as reviewed in Beltrao et al. (2012), Dewhurst et al. (2015), and Torres et al. (2016). Moreover, computational tools that quantitatively model feature-to-function relationships of PTMs for entire protein families can improve the functional prioritization of phosphorylation sites by examining each site in the context of every other family-specific PTM site that was experimentally verified (Torres et al., 2016; Dewhurst and Torres, 2017). SAPH-ire capitalizes on this benefit by inclusion of 6–8 features that have been validated to improve the functional prioritization of PTMs with known function.

While the volume of experimental PTM data from animal RGS proteins is sufficient to enable feature-to-function modeling by SAPH-ire, the relative sparsity of data observed in 7TM-RGS proteins, which are also found outside the canonical RGS family, makes this type of analysis difficult. As a result, the RGS protein family was not included in previous iterations of SAPH-ire reported in the literature (Dewhurst et al., 2015; Torres et al., 2016; Dewhurst and Torres, 2017). To overcome these limitations, we analyzed each 7TM-RGS phosphorylation site from plants in three stages. First, we analyzed the conservation of phospho-acceptor residues (S/T/Y) in the plant-specific 7TM-RGS sub-family (plant sub-family) (**Supplementary Figure S2A**). Second, we analyzed the conservation of phospho-acceptor residues in the RGS sub-family comprised of structurally resolved proteins that harbor experimentally verified PTMs (PTM sub-family) (**Supplementary Figure S3**). Third, we used SAPH-ire to compare the plant phosphorylation sites identified here with the experimental PTM sites that were contained within the PTM sub-family.

### Phosphosite Conservation within 7TM-RGS Proteins

**FIGURE 2:**
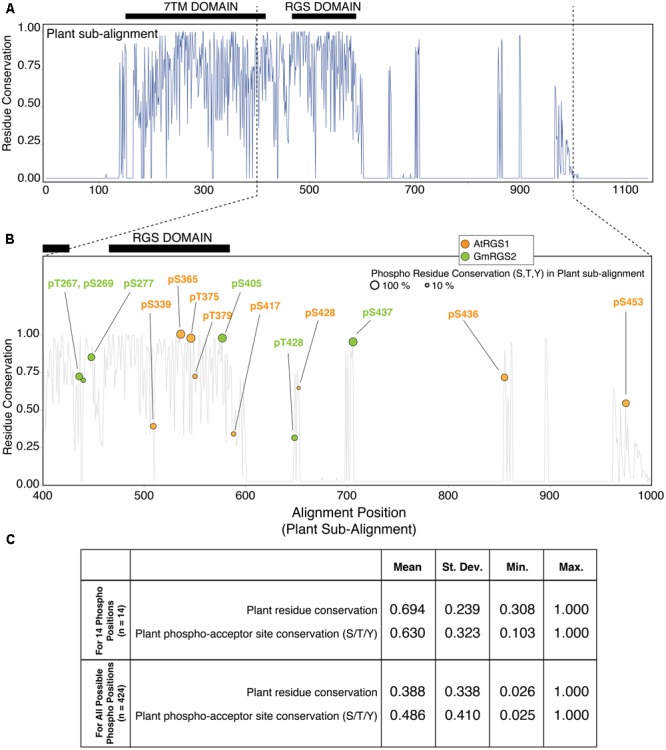
Plant phosphosites are enriched at well-conserved phospho-acceptor positions across 7TM-RGS proteins. **(A)** Residue conservation (% identity) derived from multiple sequence alignment (MSA) of 7TM-RGS proteins from plants. Black bars indicate the position of the 7TM and RGS domains. **(B)** Alignment position locations of plant phosphosites observed in the cytoplasmic C-terminal end of 7TM-RGS proteins. Only alignment positions 400–1000 are shown. Size of each circle indicates the percent conservation of phospho-acceptor residues (S,T,Y) within the alignment position of each observed phosphosite. **(C)** Table of the average residue conservation (% identity) and phospho-acceptor residue conservation (% S,T,Y) in the 14 phosphosite alignment positions (top) versus all other alignment positions that harbor at least one phospho-acceptor residue in the cytoplasmic region of 7TM-RGS.

Multiple sequence alignment of plant RGS proteins revealed high overall sequence conservation (% identity) across 7TM-RGS family members (**Figure 2A**). The alignment also conforms well with previous studies that include several additional plant sequences not yet curated by Uniprot as can be seen by direct comparison (**Supplementary Figure S2B**) (Hackenberg et al., 2016). Thus, PTM analyses performed here can be extrapolated to phylogenetically distant plant RGS groups such as *Asparagales* and *Poales* among others. The greatest degree of conservation is observed in the N-terminal half of the sub-family, corresponding to the 7TM and RGS domains that extend to alignment position 600. Beyond this position, several gaps are inserted into the MSA simply due to the C-terminal end of the green alga *Chara braunii* (a highly divergent algal species in the MSA), with interspersed well-conserved sequence islands.

We focused further analyses on the cytoplasmic region (outside the 7TM domain) which harbored all 14 plant phosphorylation sites (**Figure 2B**). Within this region, only 5 of 14 sites were located within the RGS domain itself, three of which (AtRGS1-S365, S375, and GmRGS2-S405) were located in alignment positions with 100% phospho-acceptor residue (S/T/Y) conservation. Highly conserved phosphosites were not necessarily restricted to the RGS domain, but were also found in the region between the 7TM and RGS domain as well as the C-terminal sequence islands that were well-conserved for the entire family (**Figure 2B**). Alignment positions harboring a phosphosite were also more conserved on average compared to all other alignment positions in which phosphorylation is possible (63% vs. 49%), and 8 of 14 sites were found in positions with greater than 65% phospho-acceptor residue conservation (AtRGS1-pS365, pT375, pS436, pS453; and GmRGS2-pT267, pS277, pS405, pS437) (**Figure 2C**). Thus, RLK and WNK-mediated phosphorylation is enriched at positions in which most 7TM-RGS proteins can also be phosphorylated.

### Plant Phosphosite Conservation within the PTM Sub-family

**FIGURE 3:**
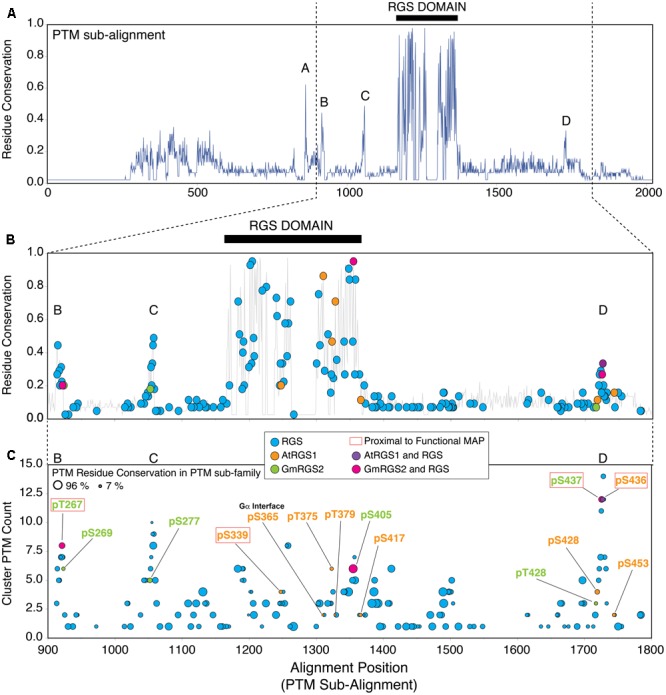
Plant phosphosites are enriched in well-conserved phosphorylation clusters observed in non-plant RGS proteins. **(A)** Residue conservation (% identity) derived from MSA of AtRGS1, GmRGS2 and all non-plant RGS proteins that harbor at least one post-translational modification (PTM) (PTM sub-family). Five well-conserved regions are observed from the alignment, including: short conserved sequence regions SCR-A, SCR-B, SCR-C, SCR-D, and the RGS domain (black bar). Positions 1–2000 are shown. **(B)** Modified alignment positions (MAPs) for all curated PTMs in the PTM sub-family of RGS proteins. Plant MAPs are included and each MAP type is color-coded as indicated in the inset. Positions 900–1800 are shown. **(C)** Plant and non-plant MAPs organized by cluster PTM count (corresponds to the PTM count observed within all MAPS ±2 alignment positions flanking the target MAP). Size of each circle indicates the percent conservation of PTM-acceptor residues within the MAP. Red boxed labels indicate that the plant MAP is proximal (±2 alignment positions) to a non-plant PTM site with a characterized biological function (see text). “RGS” MAPs correspond to MAPs harboring non-plant PTMs exclusively. 7TM-RGS proteins from plants. Black bars indicate the position of the RGS domains.

We next determined whether plant phosphosites were also well-conserved in the context of the PTM sub-family comprised of structurally resolved RGS proteins that harbor at least one PTM (**Figure 3**). To enable this comparison and preserve AtRGS1 and GmRGS2 sub-alignment, we aligned the sequence profile of the plant proteins (derived from the plant sub-alignment) to the MSA for the PTM sub-family. As a result, the relative relationship between AtRGS1 and GmRGS2 phosphosites was retained.

Sequence conservation in the PTM sub-family (% identity) is lower overall compared to the plant sub-family, with five distinct conserved regions that are dispersed along the sequence length of the alignment (**Figure 3A**). Not surprisingly, the highest degree of residue conservation was found within the RGS domain (alignment positions 1167–1367; 53% identity). In addition, four short conserved sequence regions (SCR) were also observed flanking the RGS domain including: SCR-A (alignment positions 858–864; 29% identity), SCR-B (alignment positions 912–923; 27% identity), SCR-C (alignment positions 1050–1058; 30% identity), and SCR-D (alignment positions 1721–1728; 24% identity) (**Figure 3A**). Plant-specific phosphosites were located specifically within the most well-conserved regions in the PTM sub-alignment – including SCR-B, SCR-C, SCR-D, and the RGS domain located between alignment positions 900–1800, and were not found within regions between these positions (**Figure 3B**; orange, green, purple, pink circles). In comparison, PTMs observed in non-plant proteins were broadly dispersed throughout the entire sequence length of the family (**Figure 3B**; blue circles). Thus, we conclude that RLK and WNK-mediated phosphorylation occurs in cytoplasmic regions of 7TM-RGS proteins that are more, rather than less, conserved between plants and mammals with the caveat of the ascertained bias.

To estimate the degree to which RLK- and WNK-mediated phosphorylation sites were targeted randomly, we quantified the enrichment of plant phosphosites over random expectation (see Materials and Methods). We restricted the enrichment analysis to serine and threonine (S/T) residues found within the cytoplasmic portion of the plant RGS proteins, because all mapped plant phosphosites were found on only these two residue types. In AtRGS1, 29 S/T sites were located throughout the cytoplasmic region of which eight were detectably phosphorylated *in vitro* (**Figure 1**). Five of the eight phosphosites were localized within or immediately adjacent to the RGS domain (**Figure 3B**) which harbors 13 possible sites of phosphorylation (in AtRGS1) resulting in a 1.6-fold enrichment over random expectation. One site, AtRGS1-pS436, was located precisely within SCR-D, but was not enriched beyond random expectation. Finally, the two remaining sites (AtRGS1-pS428 and AtRGS1-pS453) were close to, but outside, SCR-D and therefore not enriched within a conserved region of the sub-alignment. Unlike AtRGS1, phosphorylation of GmRGS2 was enriched in SCR-B (fourfold), SCR-C (threefold), and SCR-D (twofold), but was not enriched over random expectation in the RGS domain. Thus, phosphorylation of AtRGS1 and GmRGS2 occurs within regions that are conserved across eukaryotes and at a frequency that is higher than expected by chance. Whereas the RGS domain is the predominant target of RLK-mediated phosphorylation in AtRGS1, regions flanking the RGS domain are the predominant targets of phosphorylation in GmRGS2.

### PTM Clusters Surrounding Plant RGS Modified Alignment Positions (MAPs) Reveal Possible Phosphosite Functions

Next, we compared the 14 plant phosphosites to 155 experimentally verified PTM sites found in the RGS protein family using Structural Analysis of PTM Hotspots (SAPH-ire) – a computational informatics tool that enables functional prioritization of PTMs by compiling multiple feature-to-function relationships for modified alignment positions (a.k.a. MAPS; alignment positions that harbor at least one PTM) (Dewhurst et al., 2015). PTM data are combined by SAPH-ire into MAPs across all eukaryotic protein family members for which PTMs have been identified. MAPs are then quantitatively compared with respect to individual and/or integrated features that have been established *a priori* as positive correlates of biologically functional PTMs (Torres et al., 2016).

A total of 242 RGS family MAPs were identified by SAPH-ire, of which 76 were located in clusters within ±2 alignment positions of the 14 plant MAPs (**Supplementary Table S1**). The likelihood that a MAP is biologically functional (i.e., that a MAP harbors at least one example of a PTM that has been shown to be functional) increases proportionally with an increase in the number of PTMs found within ±2 residues of the site in question – which we defined previously as the cluster PTM count (Torres et al., 2016). Moreover, it is also well-established that functional phosphorylations are more frequently found in MAPs with conserved phospho-acceptor residues (Beltrao et al., 2012; Torres et al., 2016). We surveyed each of the 14 plant-associated MAPs (plant MAPs) with respect to cluster PTM count and modifiable residue conservation (a.k.a. PTM residue conservation – represented by circle size) from the PTM sub-alignment that includes AtRGS1 and GmRGS2 (**Figure 3C**). We observed several distinct regions that exhibit a peak cluster PTM count of four or more, most of which corresponded with SCRs B, C, D, and the RGS domain. Notably, 4 of the 14 plant phosphosites corresponded precisely with non-plant PTMs (i.e., part of the same MAP) and were also at or near the peak of PTM clusters, including: GmRGS2-pT267, AtRGS1-pS405, GmRGS2-pS437, and AtRGS1-pS436 (**Figure 3C**). In some cases, plant phosphosites were also co-incident with or in proximity to non-plant PTMs that are known to be biologically functional (**Figure 3C**, phosphorylated residues in red boxes).

**FIGURE 4:**
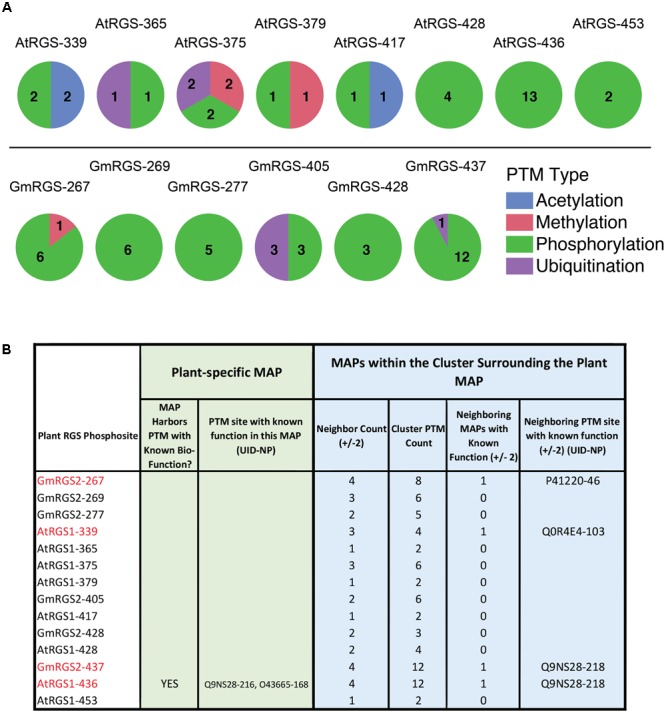
Post-translational modification cluster characteristics of plant phosphosites. **(A)** PTM types observed in PTM clusters surrounding each of the 14 plant MAPs. (top) AtRGS1-specific cluster analysis. (bottom) GmRGS2-specific cluster analysis. Numbers correspond to the number of unique PTMs found in each cluster (by PTM type). **(B)** Table showing the specific cluster characteristics for each plant phosphosite, including those within close proximity to biologically functional PTMs in non-plant RGS proteins. Neighbor count corresponds to the number of MAPs within ±2 alignment positions of the plant MAP (maximum = 4). Cluster PTM count corresponds to the total number of PTMs observed within ±2 alignment positions of the plant MAP. Neighboring MAPs with known function corresponds to a binary classifier for proximal functional PTMs (0 = NO, 1 = YES). UID, UniProt ID; NP, native position of the modified residue.

To investigate the type and density of PTM clusters surrounding each plant phosphosite, we surveyed all PTMs contained within plus and minus 2 positions surrounding each plant MAP (**Figure 4A**). We found that 8 of the 14 phosphosites were localized in PTM clusters comprised almost entirely of phosphorylation – suggesting that these sites are utilized by several different organisms. Three of these (GmRGS2-267, GmRGS2-437, and AtRGS1-436) are particularly interesting because they are part of dense phosphorylation clusters (6 count, 12 count, 13 count phospho-clusters, respectively) that harbor functional PTMs (**Figure 4B** and **Supplementary Table S2**). GmRGS2-pT267 neighbors a site in human RGS2 (P41220; S46) that is phosphorylated by cGMP-dependent protein kinase (PKGI-alpha) and necessary for activation of the RGS protein and resultant attenuation of receptor-mediated vascular contraction (Nalli et al., 2014), as well as for plasma membrane localization and control of protein degradation (Osei-Owusu et al., 2007). GmRGS2-pS437 and AtRGS1-pS436 are precisely coincident with phosphorylation or proximal to phosphorylation sites observed in human RGS18 (Q9NS28; S216 and S218) and RGS10 (O43665; S168), both of which have been established as regulatory. Phosphorylation of RGS18-S216 is stimulated by thrombin, thromboxane A2, or ADP, and promotes interaction between RGS18 and 14-3-3 proteins (Gegenbauer et al., 2013). Phosphorylation of RGS10-S168 is catalyzed by cAMP-dependent PKA and leads to inactivation of the protein by nuclear translocation (Burgon et al., 2001). Finally, AtRGS1-pS339, which does not overlap with non-plant PTMs, participates in a low density phosphorylation/acetylation cluster and is proximal to a site in rabbit RGS4 (Q0R4E4; S103) that regulates its ability to inactivate Gαq upon phosphorylation by the mitogen-activated protein kinase ERK1/2 (Mahavadi et al., 2014). Taken together, several of the RLK and WNK-mediated phosphorylation sites experimentally observed in 7TM-RGS proteins align with phosphorylation hotspots that are important for the regulation of RGS activity and protein interactions. Where GmRGS2-T267 is proximal to phosphosites that modulate GAP activity, GmRGS2-S437 and AtRGS1-S436 are coincident with phosphosites known to regulate RGS activity, protein interactions, and cellular localization. While the proximity to known-functional MAPs does not necessitate the function of phosphorylation in plant RGS, these associations provide clues to the importance of these sites in plant G protein signaling systems for experimental testing.

### Structural Topology of PTMs in the RGS Domain and Their Relationship to Plant Phosphosites

**FIGURE 5:**
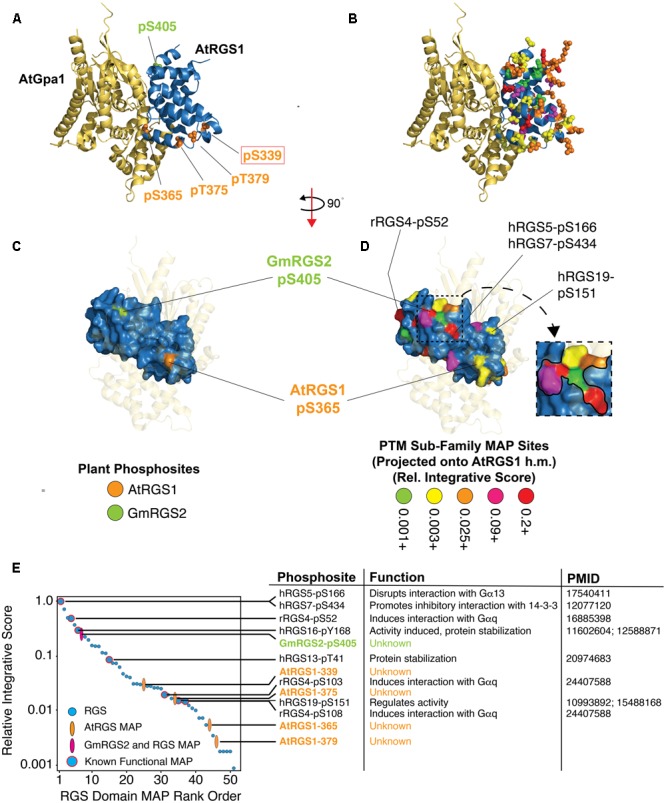
Structural projection and quantitative rank ordering of plant and non-plant MAPs identifies potential functional roles for plant phosphosites. **(A)** Structural homology model of the RGS domain of AtRGS1 bound to the crystal structure of AtGPA1 (PDB: 2XTZ) with relevant plant phosphosite sidechains rendered as Van der Waals spheres. AtRGS1 phosphosites (orange) and GmRGS2 phosphosite (green). **(B)** Same as in **(A)**, but with the projections derived from non-plant and plant MAPs in the PTM sub-family. Color code corresponds to the relative integrative score (see **E**). **(C,D)** Surface rendering of the RGS domain with corresponding structural projections shown in **(A,B)**, respectively. AtGPA1 is transparent and the structure rotated to reveal the Gα binding surface of the RGS domain. Visible RGS domain PTMs that were established as biologically functional are indicated with labels showing the RGS protein and residue position of the functional PTM. (D-inset) Zoomed view of the surface PTM cluster containing GmRGS2-pS405. **(E)** The 51 MAPs within the RGS domain were rank ordered by a relative integrative score (methods) (right). Functional PTMs contained with each MAP are shown with a brief description of their functional role and the PubMed ID source of functional evidence. (h.m.) Homology model developed using 2GTP PDB structure.

The RGS domain of RGS proteins defines the primary function of the protein family and the structure of the domain is well-characterized. Structural projection of PTM hotspots – a feature embedded within SAPH-ire – enables visualization of hotspots in the context of high-resolution X-ray crystal structures and provides meaningful context about the local three-dimensional environment of PTMs (Dewhurst et al., 2015). Using this feature, we projected both plant and non-plant RGS domain PTMs onto a structural homology model of AtRGS1 bound to its cognate Gα subunit, AtGPA1 – the high-resolution structure of which we solved previously (PDB: 2XTZ) (Jones et al., 2011) (**Figures 5A–D**). The structural homology model and orientation of this model with AtGPA1 was produced by 3-D alignment with the structure of human RGS1 bound to activated Gαi1 (PDB: 2GTP) (methods). We then determined the interface residues observed between the two proteins in the model and combined this information with the MAP feature data from SAPH-ire. As we showed previously, integrating multiple features to provide a single scoring factor enables the direct and quantitative comparison of each MAP to one another in a protein family (Dewhurst et al., 2015; Torres et al., 2016). Therefore, we created a relative integration score (IS) for each MAP in the RGS domain that included weighted and non-weighted MAP features including: PTM count, PTM residue conservation, neighbor known count, and protein interface residence defined using the homology model (methods). Each RGS domain PTM was then rank ordered in terms of its relative IS score, which was also used to color-code the structurally projected MAPs from the PTM sub-family (**Figure 5E**).

Structural projection of the four RGS domain phosphosites from AtRGS1 (pS339, pS365, pT375, pT379) and one from GmRGS2 (pS405) showed that they exist on opposite ends of the domain (**Figure 5A**). For contrast, we projected the five plant phosphosites along with 46 RGS domain PTMs from the PTM sub-alignment (i.e., PTMs from several different family members projected as color-coded cluster PTM counts), which revealed widespread coverage across most of the domain (**Figure 5B** and **Supplementary Figure S4**). Visualizing the PTM sites after surface rendering illustrates that only GmRGS2-pS405 and AtRGS1-pS365 are readily accessible (**Figures 5C,D**). AtRGS1-pS365 is not found in a dense PTM cluster and is not well-conserved in non-plant RGS proteins, indicating that this region is not commonly utilized as a regulatory phosphosite in non-plant RGS proteins (**Figure 4**). The site is also poorly conserved in plants (**Figure 2B**). However, despite this, AtRGS1-pS365 (which is conserved in GmRGS2) is located precisely at the interface modeled between AtRGS1 and AtGPA1 (**Figure 5A** and **Supplementary Table S3**). Thus, AtRGS1-pS365 may provide a more restricted but direct effect on Gα subunit interactions for AtRGS1 and GmRGS2, specifically. Other AtRGS1 phosphosites found in the RGS domain (pT375, pT379, and pS339) are somewhat buried in comparison, including pS339 that is proximal to RGS4-103, which when phosphorylated, inhibits its interaction with Gαq (Mahavadi et al., 2014). Thus, considering its distance from the RGS/Gα interface, AtRGS1-pS339 and RGS4-pS103 may function allosterically to disrupt an interaction-competent domain structure at the Gα interface.

In contrast to AtRGS1 phosphosites in the RGS domain, GmRGS2-pS405 exhibits several features that are commonly associated with functional PTMs, which can be visualized by structural projection as well as by rank ordering IS values of RGS domain MAPs (**Figures 5D,E**). Indeed, the MAP harboring pS405 ranks within the top 7 of all RGS domain MAPs, three of which are known to be biologically functional (**Figure 5E**). Moreover, pS405 is one of several MAPs that contribute to a continuous domain surface comprised of six MAPs, including one that harbors two functional PTMs (hRGS5-pS166 and hRGS7-pS434) (**Figure 5D**). Both human phosphosites regulate RGS protein interactions. Phosphorylation of hRGS5-S166 by PKC abolishes its binding capacity for Gα subunits (Moroi et al., 2007), while phosphorylation of hRGS7-S434 promotes association of the RGS protein with 14-3-3, resulting in deactivation of the domain (Benzing et al., 2002). Thus, despite a lack of alignment proximity to PTMs with known function, the structural topology of pS405 reveals that its location may alter direct or allosterically coupled interactions between the RGS domain and other proteins, including Gα subunits.

GmRGS2-pS405 may also be indirectly involved in ubiquitin-mediated regulation of the 7TM-RGS protein. Functional crosstalk between phosphorylation and ubiquitination events that control protein degradation, trafficking, among other aspects of protein regulation, are often near each other such that the probability of a functionality increases dramatically when the phosphosite is within five residues of the ubiquitination site. We found that the MAP harboring the plant phosphosite is in a PTM cluster comprised exclusively of phosphorylation and ubiquitination sites observed in human RGS10, RGS13, RGS14, and RGS19 (**Figure 4A** and **Supplementary Table S1**). Lending further support to this hypothesis, we found that GmRGS2-pS405 and AtRGS1-pS406 (which was not observed as a phosphosite, but aligns with S405) are indeed within five residues of lysine residues GmRGS2-K410 and AtRGS1-K411, both of which are 88% conserved (identical) in the plant 7TM-RGS sub-family. As the role of these proximal phosphorylation and ubiquitination sites has yet to be determined, further work will be required to establish the validity of this intriguing hypothesis.

## Conclusion

**FIGURE 6:**
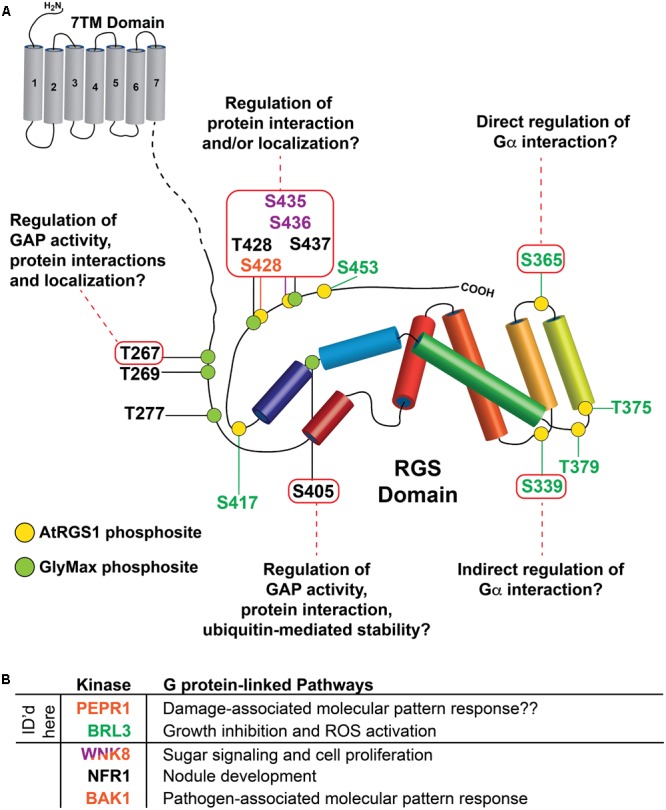
Summary of potential functions for RLK targets on plant RGS proteins identified by SAPH-ire. **(A)** Potential functional roles of indicated phosphorylation sites (inside red boxes) were derived from SAPH-ire detection and analysis of co-aligned, sequence-neighboring, or structurally proximal phosphorylation sites with reported functional effects on mammalian RGS proteins. As in **Figure 1**, phosphorylation sites identified in this study on AtRGS1 and GmRGS2 as well as sites identified previously are illustrated in the context of the domain architectures of AtRGS1 and GmRGS2 (yellow and green circles). Kinases are indicated by site ID color: BRL3 (green), BAK1, PEPR1, and WNK8 (orange) and WNK8 only (purple). NRF1 phosphorylation sites are indicated for GmRGS2 sites (black). Domain sizes are not to scale to emphasize the RGS domain and surrounding regions. **(B)** Table of kinases that phosphorylate AtRGS1 and GmRGS2 and the G protein-linked pathways in which they have been associated or are thought to be associated (indicated with “??”). Each kinase is color coded to match the phosphosites indicated in **(A)**, above. Kinases for which RGS phosphorylation sites were identified by mass spectrometry in this report are noted.

This study provides the most comprehensive profiling and analysis of plant RGS PTMs to date. Furthermore, the data are analyzed in the context of all eukaryotic RGS modification data, and thereby reveal several structural and functional PTM relationships in RGS proteins from both plants and animals. A summary of the cumulative dataset, including kinase/G protein-linked pathways and physiological responses are provided for context (**Figure 6**). By using mass spectrometry and bioinformatics approaches, we found 14 phospho-acceptor sites, three of which, GmRGS2 S267, S437 and AtRGS1 339, are suggested to be important for regulation of GAP activity, stability and localization due to their close proximity to functional mammalian RGS sites. The phosphorylation island containing the highly conserved GmRGS2 S437, T428, and AtRGS1 S428, S435/436 is a key site for regulation of RGS protein interactions or its localization. Here, we prioritized them for rigorous testing.

BAK1, PEPR1, and BRL3 each phosphorylate S428 on AtRGS1. This site is also phosphorylated by WNK8 kinase, which also phosphorylates AtRGS1 S435/436 (Urano et al., 2012). Mutations of AtRGS1 at S428 and S435/436 abolish ligand-dependent endocystosis (Urano et al., 2012; Tunc-Ozdemir et al., 2016). While AtRGS1 S435/436 is a well-conserved site, S428 is not except in closely related *Brassica napus, Cleome hassleriana*, and *Erythranthe guttata*. However, GmRGS2 pT428, which aligns closely to AtRGS1 pS428, is more conserved. This suggests S428 and T428 might be used interchangeably. Another possibility is that this is a phosphorylation island including the highly conserved GmRGS2 T428, S437, and AtRGS1 S435/436. Therefore, the function of all the phosphorylation sites in this region is likely regulation of RGS protein interactions or its localization for the reasons discussed above.

In addition, pS405 of GmRGS2 are near conserved phosphorylation sites of human RGS5 (S166), RGS7 (S434), and RGS16 (Y168). Phosphorylation of Ser166 in RGS5 by protein kinase C causes loss of RGS function. RGS5 protein phosphorylated by PKC showed much lower binding capacity for and GAP activity toward Gα subunits than did the unphosphorylated RGS5 (Moroi et al., 2007). The phosphorylation-dependent interaction of 14-3-3 with RGS7 inhibits its GTPase-accelerating activity *in vitro*. Tumor necrosis factor TNF-α, which is a cell signaling protein (cytokine) involved in systemic inflammation, reduces serine S434 phosphorylation of RGS7 and the interaction of RGS7 with 14-3-3 (Benzing et al., 2002). Phosphorylation of RGS16 conserved tyrosine residue (Y168) in the RGS box by src kinase increases GAP activity (Derrien and Druey, 2001). Phospho-mimetic mutant (GmRGS2^S405D^) did not change GAP activity *in vitro* (Choudhury and Pandey, 2015) but this could be due to lack of a protein like 14-3-3 in the assay. Due to its close proximity to all these conserved sites, pS405 is most likely essential for RGS function and controls the GAP activity through RGS interaction with other proteins like 14-3-3.

## Author Contributions

MT-O, DU, AJ, and MT designed the experiments; MT-O, BL, and DJ performed the *in vitro* phosphorylation experiments and MT analyzed the results via SAPH-ire; and MT-O, AJ, and MT wrote and approved the manuscript.

## Conflict of Interest Statement

The authors declare that the research was conducted in the absence of any commercial or financial relationships that could be construed as a potential conflict of interest.

## Supplementary Material

FIGURE S1 Detailed optimization process for BRL3 autophosphorylation and AtRGS1 phosphorylation assay and mass spectrometry peptide coverage. **(A)** MgCl_2_ and MnCl_2_ concentrations were changed from 0 to 10 mM in reaction buffer where purified BRL3 kinase domain was mixed with 6XHis-tagged AtRGS1 C-terminal domain (His6-RGS-J5) protein. Even though differences observed within different combinations were small, 10 mM MgCl_2_ with 5 mM MnCl_2_ was chosen as the best condition for the reactions used for Mass Spectrometry analysis. **(B)** *In vitro* kinase assay of PEPR1 phosphorylation of AtRGS1 and mass spectrometry peptide coverage.

FIGURE S2 Phylogenetic tree of the plant sub-family. **(A)** Evolutionary relationship of all 7TM-RGS proteins included within this study. **(B)** Analysis of residue identity (percent) at alignment positions shown previously to be well-conserved across phylogenetically distant plant species that have not yet been curated in Uniprot (based on Hackenberg et al., 2016). Alignments used here included uniprot-curated data only.

FIGURE S3 Phylogenetic tree of the PTM sub-family. Evolutionary relationship of all non-plant RGS proteins included within this study.

FIGURE S4 Full rotation view of plant and non-plant MAPs projected onto the homology model of the AtRGS1 RGS domain. **(A,B)** Same as in **Figure 5**, shown here for orientation. **(C–F)** Various rotation positions that allow full visualization of all RGS domain MAPs analyzed by SAPH-ire. MAPs harboring PTMs with biological function are labeled with the specific protein and position for the modified residue.

TABLE S1 Comprehensive table of PTMs found in clusters around plant phosphosite MAPs.

TABLE S2 Comprehensive table of plant phosphosite features derived from the plant and PTM sub-alignments.

TABLE S3 Table of MAPs found in the RGS domain.
