# Supplementary figures and images for: Predicted Functional Implications of Phosphorylation of Regulator of G Protein Signaling Protein in Plants
Source: Front Plant Sci. 2017 Aug 25;8:1456. doi: 10.3389/fpls.2017.01456 (PMC5575782; doi:10.3389/fpls.2017.01456)

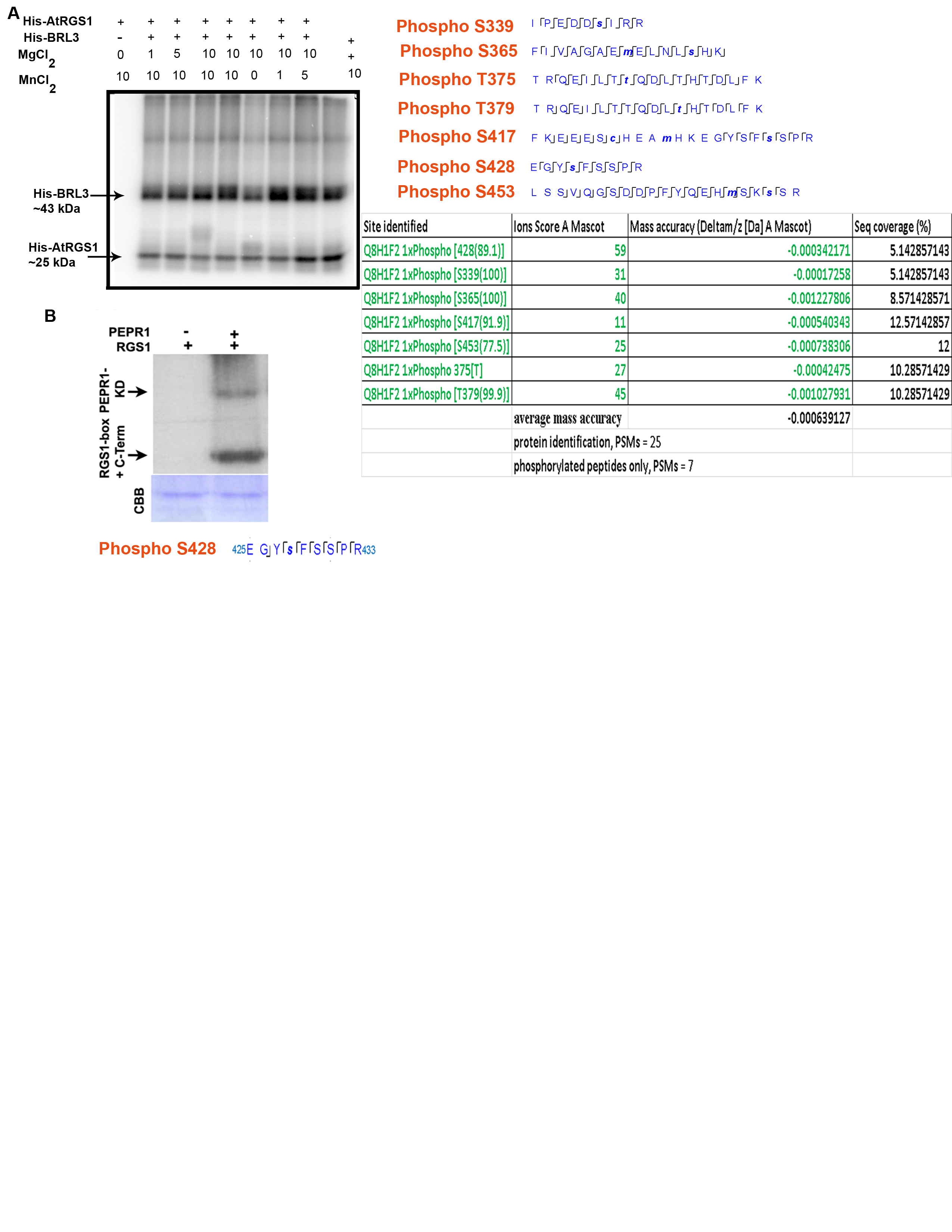

Supplement: FIGURE S1 — Detailed optimization process for BRL3 autophosphorylation and AtRGS1 phosphorylation assay and mass spectrometry peptide coverage. (A) MgCl2 and MnCl2 concentrations were changed from 0 to 10 mM in reaction buffer where purified BRL3 kinase domain was mixed with 6XHis-tagged AtRGS1 C-terminal domain (His6-RGS-J5) protein. Even though differences observed within different combinations were small, 10 mM MgCl2 with 5 mM MnCl2 was chosen as the best condition for the reactions used for Mass Spectrometry analysis. (B) In vitro kinase assay of PEPR1 phosphorylation of AtRGS1 and mass spectrometry peptide coverage. [file Image_1.JPEG]

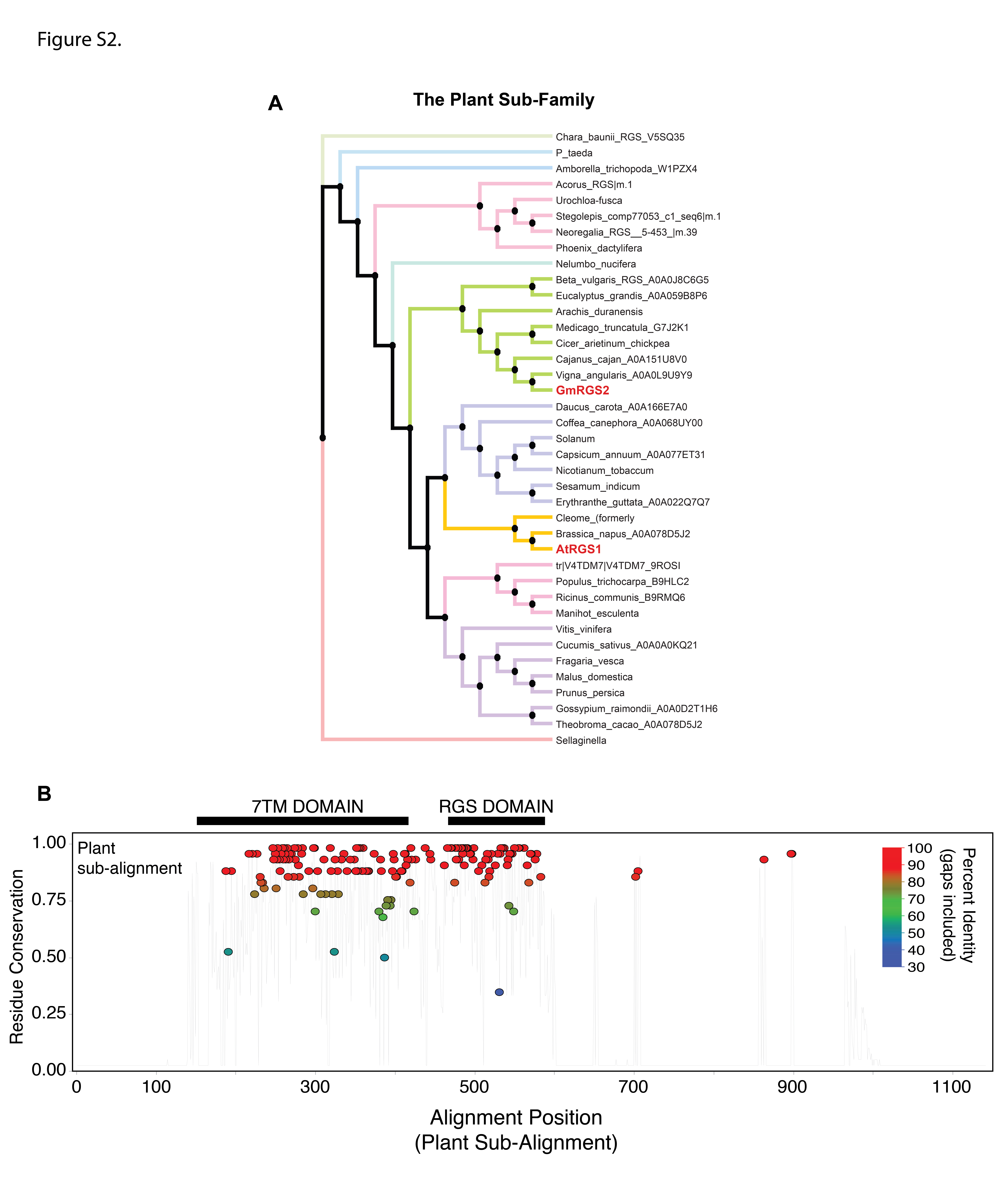

Supplement: FIGURE S2 — Phylogenetic tree of the plant sub-family. (A) Evolutionary relationship of all 7TM-RGS proteins included within this study. (B) Analysis of residue identity (percent) at alignment positions shown previously to be well-conserved across phylogenetically distant plant species that have not yet been curated in Uniprot (based on Hackenberg et al., 2016). Alignments used here included uniprot-curated data only. [file Image_2.JPEG]

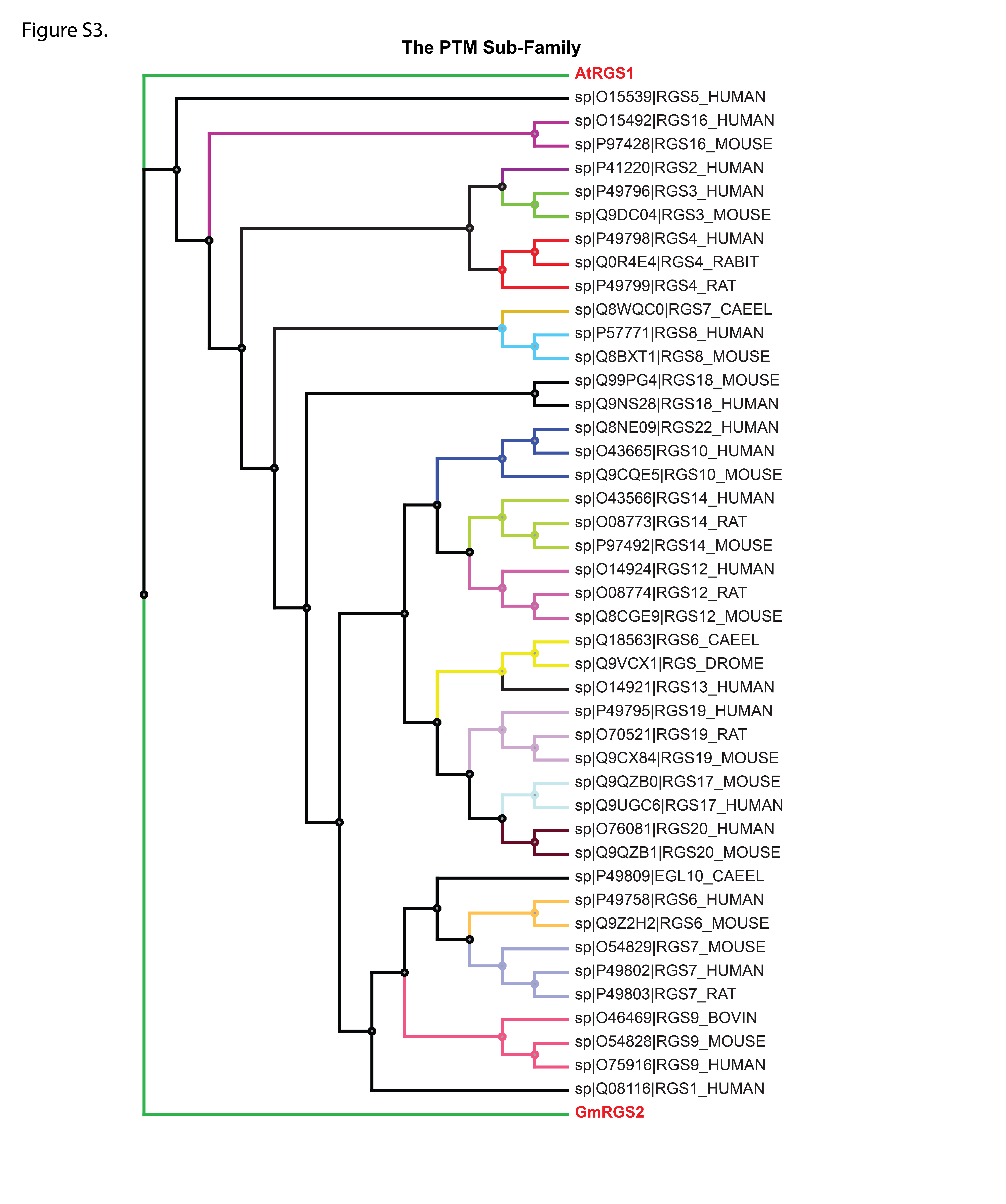

Supplement: FIGURE S3 — Phylogenetic tree of the PTM sub-family. Evolutionary relationship of all non-plant RGS proteins included within this study. [file Image_3.JPEG]

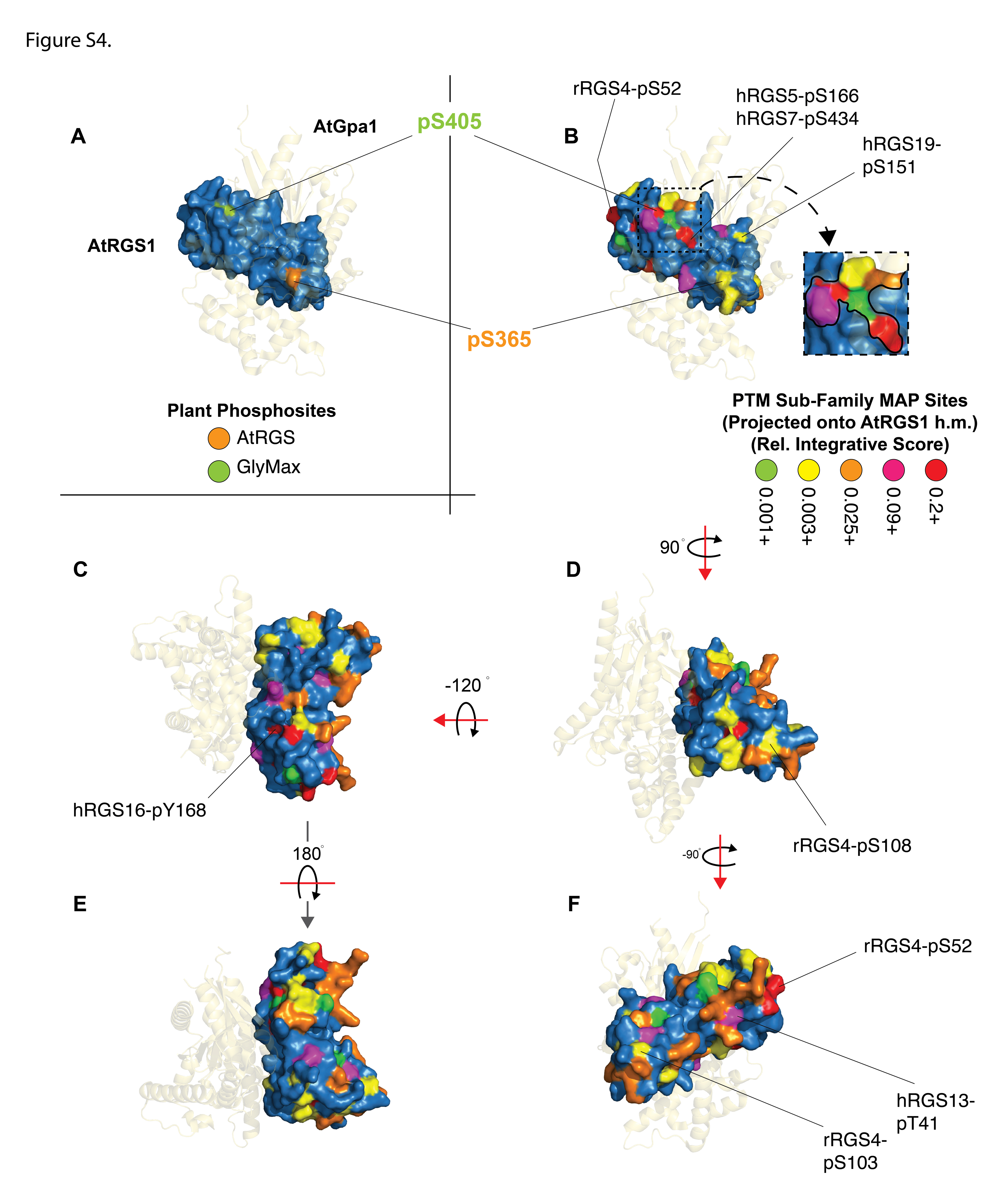

Supplement: FIGURE S4 — Full rotation view of plant and non-plant MAPs projected onto the homology model of the AtRGS1 RGS domain. (A,B) Same as in Figure 5, shown here for orientation. (C–F) Various rotation positions that allow full visualization of all RGS domain MAPs analyzed by SAPH-ire. MAPs harboring PTMs with biological function are labeled with the specific protein and position for the modified residue. [file Image_4.JPEG]
